# Effects of feeding frequency on juvenile Chinese sturgeon *Acipenser sinensis*

**DOI:** 10.1038/s41598-020-74120-x

**Published:** 2020-10-15

**Authors:** Yacheng Hu, Kan Xiao, Jing Yang, Xueqing Liu, Binzhong Wang, Qingkai Zeng, Hejun Du

**Affiliations:** 1 Chinese Sturgeon Research Institute, China Three Gorges Corporation, Yichang, 443100 Hubei China; 2 Hubei Key Laboratory of Three Gorges Project for Conservation of Fishes, Yichang, 443100 Hubei China

**Keywords:** Gene expression, Gene regulation

## Abstract

In this study, the effects of different feeding frequencies on the growth and the expression of genes in the GH/IGF axis were assessed in juvenile Chinese sturgeon. The newly hatched Chinese sturgeons were bred for 38 days at three different feeding frequencies groups (feeding frequency of two times a day, TWD; three times a day, THD; and four times a day, FOD), and the expression levels of the GH/IGF axis responses to feeding frequency were determined by quantitative real-time PCR. In addition, the full-length of the Coding Sequences of IGF I and IGF II genes (489-bp and 660-bp, respectively), were cloned and analyzed from Chinese sturgeon the first time. Multiple sequence alignments of IGFs revealed that Chinese sturgeon are high sequence identity to IGFs from other species. The phylogenetic relationships based on the IGF I and IGF II amino acid sequences were consistent with the traditional classification. After 38 days of growth, the three different feeding frequencies groups of Chinese sturgeon had no significant difference of body length, body weight, specific growth rate, the survival rate, the rate of weight gain and the condition factor. However, the relative expression of Chinese sturgeon GH in the pituitary decreased with increasing feeding frequency. The relative expression of Chinese sturgeon GHR in liver and skeletal muscle was deceased with increasing feeding frequency, while the relative expression of GHR in stomach and intestines at THD group was significantly higher than that of at TWD group and FOD group (*p* < 0.05). The relative expression of Chinese sturgeon IGF I in liver increased significantly with increasing feeding frequency (*p* < 0.05). The relative expression of IGF I in stomach and skeletal muscle was similar at the three groups. The relative expression of IGF I in intestines was significantly higher at FOD group than at TWD group and THD group (*p* < 0.05). The relative expression of Chinese sturgeon IGF II in liver at TWD group was significantly higher than that at THD group and FOD group (*p* < 0.05). However, the relative expression of IGF II in stomach, intestines and skeletal muscle at THD group was higher than that at TWD group and FOD group. Based on these previous studies that liver IGF I is regarded as a biomarker of growth performance, this result suggested that the juvenile Chinese sturgeon is better for growth when feeding four times daily compared to twice and thrice daily.

## Introduction

Chinese sturgeon *Acipenser sinensis* is an anadromous species that only spawns in Yangtze River of China^1^. The number of the wild population of Chinese sturgeon has greatly declined because of overfishing and damming^2^. And this species has been classified as a critically endangered species by the International Union for Conservation of Nature (IUCN)^3^. The wild population of Chinese sturgeon did not spawn for the first time in 2013^4^. So, it is urgently to increase the efforts of research and conservation and to protect the spawning environment of Chinese sturgeon. In this situation, artificial propagation is one of the effective strategies to enhance the wild population. To be able to develop an aquaculture industry and to save this species more efficiently, artificial reproduction has been attempted since 1983. However, it is difficult to breed Chinese sturgeon because of its life history characteristics that is long lifespan and late maturation. For Chinese sturgeon, it needs at least 14 and 9 years for females and males to grow mature^5^. Therefore, it is important to study Chinese sturgeon to speed up the rate of growth of this species. However, because of its extremely late sexual maturation which needs more than 9 and 14 years for males and females, respectively, it is very difficult to accomplish artificial reproduction. Moreover, there is little knowledge about the regulation mechanism of growth of Chinese sturgeon.

The growth of Chinese sturgeon is largely governed by food intake, feeding frequency, ration, the kind of food and its ability to absorb nutrients. Notwithstanding the nutritionally balanced feeds play an important role in cultivating fish, an appropriate feeding management is also necessary to improve production efficiency. It is well known that feeding frequency is one of the most important factors in fish farming. Feeding frequency plays important role in the growth of fish, especially in the early stages^6^. For aquaculture producers, it is critical to minimize production costs in a global market place. The appropriate feeding frequency not only increase feed conversion ratio to minimize feed wastage and to improve the water quality but also enhance growth to reduce the size heterogeneity of fish^7^. Not appropriate feeding frequency may lead to increased cannibalism or enhance costs of production^8^. Higher feeding frequency is better to feed younger age groups on higher growth and survival^9^. However, over-feeding frequency can input costs and accumulate waste that can affect water quality. Meanwhile, a too low feeding frequency may cause greater variety in fish size and reduced growth^10^. A lot of studies have done about feeding frequency. Many studies had proved that there is an optimal feeding frequency to feed fish not only for food ingestion and digestion, but also absorption occurring. Feeding five times a day resulted in exhibited improved growth rates compared with once or thrice feeding per day in juvenile Atlantic Halibut *Hippoglossus hippoglossus*^11^. Therefore, investigation of the appropriate feeding frequency for individual growth is important for successful culture of Chinese sturgeon. Chinese sturgeon culturing methods of breeding to gain higher productivity and survival rates have been developed. However, no studies have done to establish the optimum feeding frequency on Chinese sturgeon. It is important to explore the molecular mechanisms to feeding frequency to ensure optimal health of Chinese sturgeon.

The growth of fish is a complex metabolic process and physiological that is primarily controlled by the growth hormone (GH)/insulin-like growth factors (IGFs) axis^12^. The GH-IGF axis that is involved in many metabolic pathways and physiological processes related to somatic growth, development, metabolism, and reproduction plays an important role in the regulation of the growth-promoting actions in fish^13–15^. The GH/IGF axis includes GH and IGFs. The growth of fish is primarily controlled by the GH and IGFs^12,16^. Studies on the function of GH/IGFs in fish are less than those in mammalian vertebrates.

The pituitary secretes GH, which stimulates the synthesis and secretion of IGF I^17^. The GH receptor (GHR) play essential role in this process. When GH binds to GHR on the cell surface of the target tissue, it will form a GH-GHR complex. GHR play essential role in the production and release of IGF I in the liver and other tissues^18^.

GH should be considered to be the most important because its regulation involves a complex series of interactions between different hormones. GH is an essential polypeptide required for the development and growth of fish. GH is involved in carbohydrate metabolism, reproduction, skeletal and soft tissue growth, immune function and so on^19^.

IGFs plays an important role in variety cellular processes such as metabolic regulation, cell growth, organ differentiation and basic metabolism^20,21^. IGFs include IGF I and IGF II. In fish, both IGF I and IGF II are detected in liver, gastrointestinal tract, skeletal muscle, gills, brain, heart, eye, spleen, gonads, kidney and pancreatic^22,23^. IGF I played an important role in the rule of the growth-promoting actions in fish^13^. IGF I was so important in regulation growth and reproduction that this gene had been studied widely including the Southern flounder (*Paralichthys lethostigma*)^24^, the Chilean flounder (*Paralichthys adspersus*)^25^, tilapia (*Oreochromis mossambicus*), rainbow trout (*Oncorhynchus mykiss*), coho salmon (*Oncorhynchus kisutch*), carp (*Cyprinus carpio*), red seabream (*Pagrus major*)^26^. IGF I regulates most of the somatotropic activities such as carbohydrate metabolism and cellular growth^17^. IGF I was not only involved in promoting cell differentiation processes^27,28^, cell proliferation^29,30^, but also vary plastically in response to artificial selection for growth rate^31,32^ and environmental cues, such as temperature, nutrition and photoperiod^33–35^. The mRNA expression of IGF I can be elevated by intraperitoneal injection of recombinant bream GH^36^. Although a lot of knowledge of physiological functions of IGF I have been known, there is little about those of IGF II. The expression of IGF II is also influenced by GH, and IGF II is also abundantly produced in the liver and extra liver tissues^37,38^, Several studies have reported that GH dependent expression of IGF II in fish^39,40^. The IGF II may play an important role in embryonic growth in mammals^41^. However, the expression of IGF I and IGF II are by way of a negative feedback control pituitary GH expression in vitro^42^.

The mRNA transcripts of GH and IGFs in fish are considered to be an indicator for growth performance and physiological status^43,44^. A lot of studies indicate that the nutrients, seasons and developmental can affect the level of IGFs in fish^45–47^. For instance, the Atlantic salmon had a significant decrease in circulating IGF I and IGF II after prolonged starvation^48^. While, prolonged starvation led to reductions in GH mRNA expression and liver IGF I mRNA expression in *Ictalurus punctatus*^49^ and the dietary protein composition was reported to be closely correlated with the expression levels of GH and IGF I in *Carassius auratus*^50^. Thus, the GH/IGF axis was speculated to be played an important role in fish under different physiological status and environment conditions. Although IGFs are so important, there is no study on IGFs in Chinese sturgeon. And information on the GH/IGF axis in Chinese sturgeon is rare. As the primary growth related gene, understand the mechanism of GH/IGF axis will improve our knowledge on fish growth.

Thus, the objectives of our study were to explore the roles of GH, GHR and IGFs under different feeding frequencies and elucidate the optimal feeding frequency to feed juvenile Chinese sturgeon. We also cloned and analyzed IGF I and IGF II from Chinese sturgeon the first time. The knowledge gained from this research will be useful for the development of Chinese sturgeon breeding.

## Material and method

### Exprimental procedures

The fertilized eggs of Chinese sturgeon were obtained from Chinese Sturgeon Research Institute, Hubei Provence, China. They were hatched at 18.0 ± 0.5 °C. After 5 days, the eggs were hatched into larvae. After eighteen days growth of larvae, Chinese sturgeon (body weight, 0.15 ± 0.02 g; length, 3.47 ± 0.06 cm) began to eat food. Then, a total of 450 apparently healthy Chinese sturgeon individuals were randomly selected for the experiment. The 450 Chinese sturgeon individuals were randomly stocked into 9 rectangle tanks (0.8 m × 0.5 m × 0.4 m) at 18.0 ± 0.5 °C (50 fish in each tank). The experiment was partitioned into three groups. Among the 9 rearing tanks of fish, three were fed two times daily with Red silk worm (TWD), another three were fed three times daily (THD), the last three were fed four times daily (FOD), each group was set up with 3 parallel tanks, and there are 50 fish in each tank. The dissolved oxygen of the water in the tanks was maintained at over 6.0 mg/L throughout the experiment. The tanks were supplied with filtered water. The physicochemical parameters of the water in the tank can meet the need of the experiment. In our study, stocking density was not a limiting factor during the experiment, because the tank was large enough to meet the experiment requirements. In addition, before the main experiment, a pre-experiment was performed to determine bout/meal size and time spent eating (time in minutes), and the result showed that feed time was completed within ~ 40 min. Therefore, during the experiment, Chinese sturgeon was used apparent satiety feeding method to be fed with enough red silk worm each time, and residues were cleared after 40 min of feeding. The specific feeding time in each day is at 9:00 and 21:00 in TWD group, 9:00, 15:00 and 21:00 in THD group, 9:00, 13:00, 17:00 and 21:00 in FOD group. All rearing tanks were changed clean water two times a day to maintain the water quality. The light regime was controlled at 12 h light and 12 h dark. They were fed 38 days. Body weight and length of body were measured at the beginning and the end of experiment. Then six fish was taken from each rearing tanks to be sampled. All fish were anaesthetized with MS-222 (Sigma, USA) and sampled within 5 min. Pituitary, liver, stomach, intestines and skeletal muscle were collected from each fish, immediately frozen in liquid nitrogen, and stored at − 80 °C.

### Measurement of growth traits

The initial body weight (IBW) (g), initial body length (IBL) (cm), finally body weight (FBW) (g) and finally body length (FBL) (cm) was measured at the beginning and end of the trial. Specific growth rate (SGR) was calculated as follows: SGR (%) = 100 × (lnFBW − lnIBW)/time (d). The survival rate (SR) was calculated as follows: SR = 100% × N_f_/N_i_, N_f_ is the number of the finally fishes, N_i_ is the number of the the initial fishes. The rate of weight gain (WGR) was calculated as follows: WGR = 100% × (FBW-IBW)/IBW. The condition factor (CF) was calculated as follows: CF = 100% × FBW/FBL^3^.

### Molecular cloning of IGFs and GHR cDNAs

Total RNA was extracted from the samples using Trizol reagent (Invitrogen, USA) according to the manufacturer’s instructions. The concentration of total RNA and the purity of sample were quantified by NanoDrop®2000 spectrophotometer (Thermo Scientific, Wilmington, DE, USA) and 1% agrose gel. All RNA samples analyzed had a 260/280 ratio ≥ 1.80. The cDNA was produced following the manufacturer’s instructions (iScript cDNA Synthesis Kit, BioRad, Hercules, CA, USA) by using one microgram of RNA. Then the cDNA was stored at − 80 °C to be used as a template in quantitative real-time PCR (qPCR).

Based on unpublished Chinese sturgeon transcriptome sequences (Illumina HiSeq2000) that was measured by our laboratory, we obtain the partial cDNA sequences of GHR, IGF I and IGF II. The fragment-deduced amino acid sequences were most similar to GHR and IGFs protein. To obtain the full-length Coding sequence of IGF I and IGF II in Chinese sturgeon, special primers were designed according to the partial cDNA sequence that with Primer 5.0 (showed in Table 1). Then, cDNA fragments of GHR, IGF I and IGF II of Chinese sturgeon were obtained by PCR amplification. The PCR reaction was performed in a final volume of 50 μL containing 2 μL cDNA, 4 μL 10 mmol/L dNTP mix, 5 μL reaction buffer, 1 μL each primer solution (Table 1), 0.4 μL Taq polymerase (TaKaRa), and 36.6 μL nuclease-free water. All PCRs were performed on a PTC-200 thermal cycler (Bio-Rad, USA). Denaturation at 94 °C for 3 min was followed by 40 cycles of amplification at 94 °C for 30 s, annealing temperature of each gene for 30 s, and 72 °C for 30 s, followed by an additional extension at 72 °C for 10 min. The PCR products were electrophoresed on a 1.5% agarose gel and visualized using ethidium bromide staining. Putative gene fragments were cloned into pMD18-T vector (Takara, Japan) after purification, and sequenced with an ABI3730XL sequencer (Applied Biosystems, Foster City, CA, USA).

**Table 1:** Summary of quantitative real-time PCR primers used in this study.

Primers	Sequence (5′–3′)	Production size (bp)
IGF I-F1	GTGTTCTGTGCCTGACTC	402
IGF I-R1	GAAGAGCAAAATCCAGA	
IGF I-F2	CCCATTCCTTCTTATTTCGC	318
IGF I-R2	CATAGCCTGTTGGTTTGTTGA	
IGF II-F	AATGCCACAGAAAGGACG	820
IGF II-R	GCAGCAGAAACTGGGAAC	
GHR-F	TGAACATCAAGGACGACG	318
GHR-R	CGGGCGATAGCAGAAC	
IGF I-qF	GTACTGTGCGCCTGTTAA	164
IGF I-qR	TCCTGTTGCCTATGTTCC	
IGF II-qF	ATCGCCCTCACAGTCTACAT	130
IGF II-qR	GTGGCTTGCTGAAATAAAA	
GH-qF	ATGGCATCAGGTCTGCTTCT	185
GH-qR	ACGCTGCTCATCTGGAACATAG	
GHR-qF	CATAGAAATCCAGGTTTACCCAACTC	271
GHR-qR	CTGAACATCAAGGACGACGACTC	
*β*-Actin-F	TTATGCCCTGCCCCACGCTATC	201
*β*-Actin-R	CGTGTGAAGTGGTAAGTCCGT	

### Sequence analysis

To examine the similarity of Chinese sturgeon IGF I and IGF II to those of other species, the cDNA and deduced amino acid sequences were analyzed by the BLAST program (NCBI, https://www.blast.ncbi.nim.nih.gov/Blast.cgi). Multiple sequence alignments of predicted amino acid sequences of IGF I and IGF II were produced using DNAMAN software. A phylogenetic tree based on IGF I and IGF II amino acid sequences was constructed using the neighborjoining method on Mega 5.1 using 1000 bootstrap replicates. Signal peptide regions, conserved domains, protein phosphorylation site, transmembrance helix structure, secondary structure, tertiary structure, hydrophobicity prediction and protential N-glycosylation sites were predicted using SignalP version 3.0 (https://www.cbs.dtu.dk/services/SignalP/), Pfam (https://pfam.xfam.org/search), DISPHOS1.3 (https://www.dabi.temple.edu/disphos/), TMHMM Server v.2.0 (https://www.cbs.dtu.dk/services/TMHMM/), PSIPRED 4.0 (https://bioinf.cs.ucl.ac.uk/psipred/), Chimera (https://www.cgl.ucsf.edu/chimera/), ProtScale (https://web.expasy.org/protscale/) and ExPASy Molecular Biology Server (https://www.expasy.org/).

### Quantitative real-time RT-PCR (qRT-PCR)

Chinese sturgeon *β*-actin (Table 1) was used as a positive control for the RT-PCR analysis to determine the template concentration and to provide an external control for PCR reaction efficiency under the same reaction conditions. The GH sequence of Chinese sturgeon was got from in Genbank (Accession No. EU599640).

The expression patterns of GH/IGF axis mRNAs in various tissues of Chinese sturgeon were examined by RT-PCR. The qPCR were performed in triplicate wells using SYBR Green Premix Ex TaqTM II (Takara) on an AB Step One Plus Real-Time PCR system (AB Applied Biosystems, Foster City, USA) and the cycling conditions for qPCR were as follows: 95 °C for 30 s, 40 cycles of 5 s at 95 °C, 30 s at 60 °C, and 45 s at 72 °C. The primers used in this study were designed based on the cloned sequences using Primer5 software. Table 1 lists the primers used for these analyses. The relative quantification (RQ) was calculated using the 2^−ΔΔCt^ method (ΔCT = CT of target gene—CT of *β*-actin, ΔΔCT = ΔCT of any sample -ΔCT of calibrator sample).

### Statistical analysis

All data were performed using SPSS 17.0 and shown as mean values ± standard error. Statistical analyses were determined by one-way analysis of variance (ANOVA) using Duncan’s test. Significant differences were accepted when *p* < 0.05.

All fish handling and experimental procedures in this study were approved by the Chinese Sturgeon Research Institute, the China Three Gorges Corporation, and the Hubei Key Laboratory of Three Gorges Project for Conservation of Fishes. All experiments were performed in accordance with relevant guidelines and regulations.

## Results

### Effects of feeding frequency on growth performance

Table 2 summarizes the growth parameters of Chinese sturgeon after 38 days of culture. No fish died in the three different feeding frequencies group during the 38 days of experiment. After 38 days of growth, the three different feeding frequencies groups of Chinese sturgeon had no significant difference of body length, body weight, specific growth rate, the survival rate, the rate of weight gain and the condition factor.

**Table 2:** Growth parameters of Amur sturgeon after 38 days of growth at different stocking frequencies.

Item	The group of feeding two times daily (TWD)	The group of feeding three times daily (THD)	The group of feeding four times daily (FOD)
Initial body weight (IBW, g)	0.15 ± 0.02^a^	0.15 ± 0.02^a^	0.15 ± 0.02^a^
Initial body length (IBL, cm)	3.47 ± 0.05^a^	3.46 ± 0.04^a^	3.47 ± 0.06^a^
Finally body weight (FBW, g)	2.44 ± 0.5^a^	2.58 ± 0.59^a^	2.54 ± 0.49^a^
Finally body length (FBL, cm)	7.01 ± 0.56^a^	7.17 ± 0.62^a^	7.13 ± 0.51^a^
Specific growth rate (SGR)	7.51 ± 0.56^a^	7.65 ± 0.66^a^	7.64 ± 0.54^a^
Survival rate (SR) (%)	100	100	100
The rate of weight gain (WGR) (%)	1545.72 ± 336.78^a^	1644.83 ± 396.77^a^	1618.39 ± 330.92^a^
Condition factor (CF)	0.007 ± 0.00065^a^	0.0069 ± 0.00052^a^	0.007 ± 0.00055^a^

Values not sharing a common letter are significantly different (*p* < 0.05). Data are means ± SEM.

### Molecular characterization and phylogenetic analysis IGF I and IGF II

**Figure 1:**
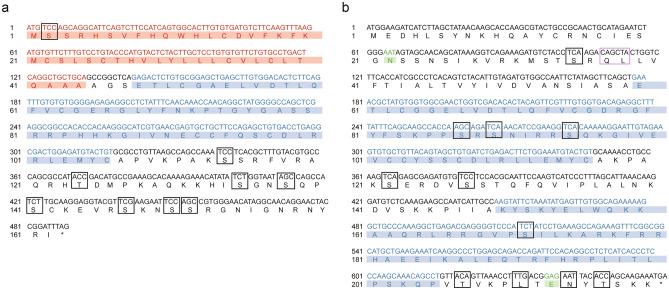
Nucleotide and deduced amino acid sequence of Chinese sturgeon IGFs. (**a**) Chinese sturgeon IGF I. (**b**) Chinese sturgeon IGF II. The putative signal peptide is marked in red. A putative conserved domain is marked in blue. Predicted phosphorylation sites are boxed in black. Prediction of glycosylation site of proteins are indicated in green. Prediction of transmembrane helix structure of protein are boxed in purple. The stop codon is denoted by an asterisk.

**Figure 2:**
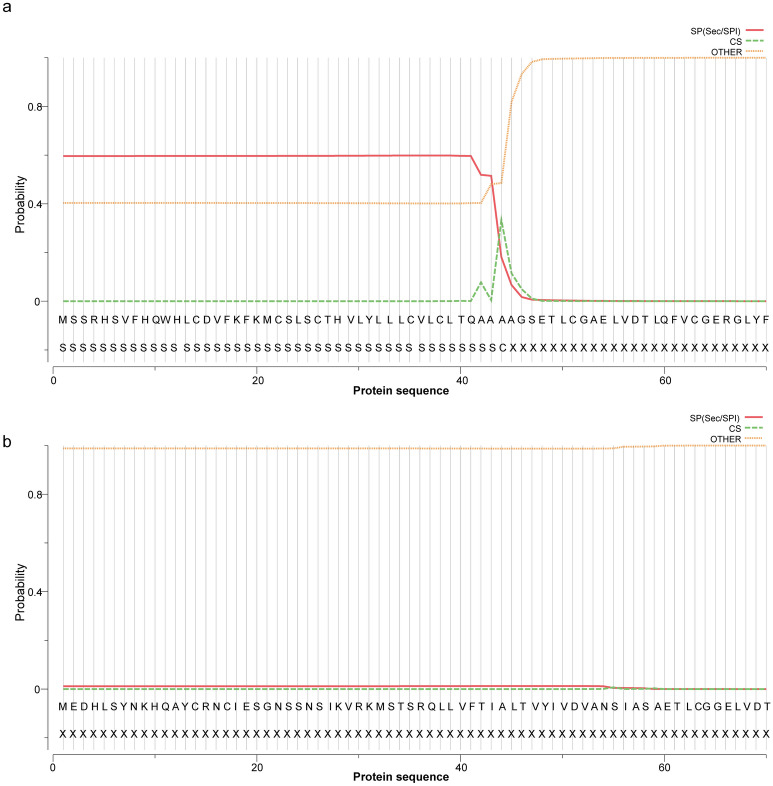
Predicted signal peptide of IGFs protein in Chinese sturgeon. (**a**) Chinese sturgeon IGF I. (**b**) Chinese sturgeon IGF II.

**Figure 3:**
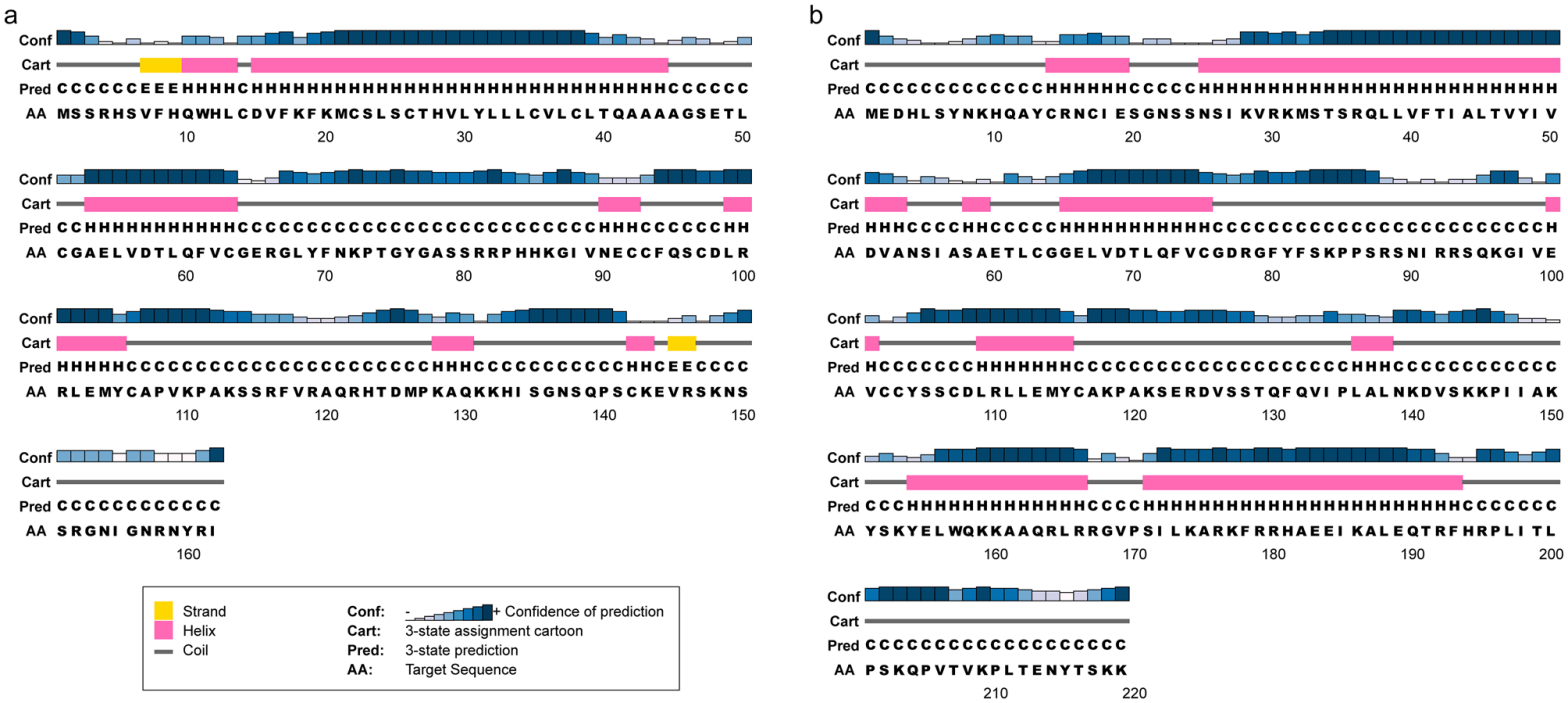
Chinese sturgeon IGFs protein second structure prediction via PSIPRED. (**a**) Chinese sturgeon IGF I. (**b**) Chinese sturgeon IGF II.

**Figure 4:**
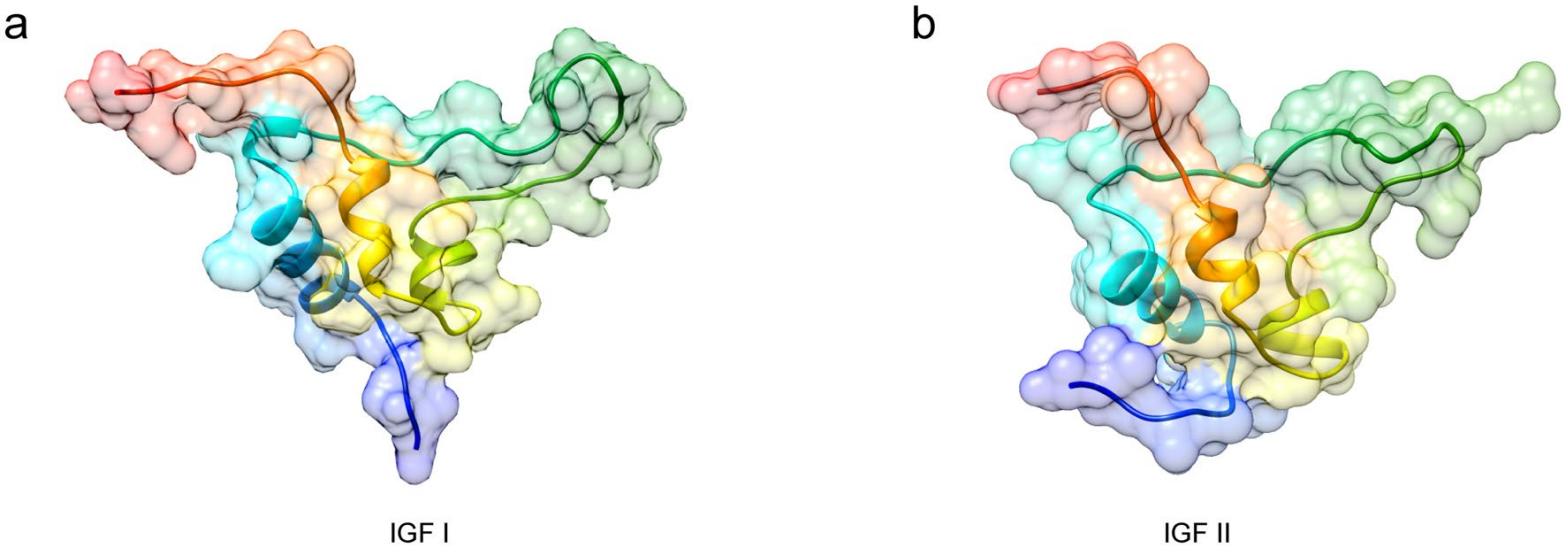
Chinese sturgeon IGFs protein tertiary structure prediction via Chimera. (**a**) Chinese sturgeon IGF I. (**b**) Chinese sturgeon IGF II.

We cloned the full-length of the Coding Sequences of IGF I and IGF II genes from the liver of Chinese sturgeon (accession no. MK028132 and MK028133). And we cloned the partial-length of the Coding Sequences of GHR gene from the liver of Chinese sturgeon (accession no. MK028131). The Coding Sequences of IGF I and IGF II genes were 489-bp and 660-bp, respectively. As shown in Fig. 1 the predicted amino acid sequence of IGF I and IGF II consist of 162 and 219 amino acid, respectively. The calculated molecular weight of IGF I protein was 18.3 kDa with a theoretical isoelectric point of 9.5. Similarly, the calculated molecular weight of IGF II protein was 25.0 kDa with a theoretical isoelectric point of 9.77. The GRAVY (grand average of hydropathicity) results indicated that IGF I and IGF II were hydrophilic. The IGF I protein contained a conserved domain (48-106aa) and nine putative phosphorylation sites. The IGF II protein contained two conserved domains (60-116aa and 150-205aa) and 11 putative phosphorylation sites. The IGF I was predicted to contain no glycosylation site and transmembrane helix structure. The IGF II was predicted to contain two glycosylation sitea and one transmembrane helix structure. The IGF I was predicted to contain 44 amino acid signal peptide, however, there is no amino acid signal peptide was predicted in the IGF II (Fig. 2). The second structure of IGF I protein was predicted to consititute with strand (3%), helix (37%) and coil (60%) (Fig. 3). The second structure of IGF II protein was predicted to consititute with helix (43%) and coil (57%) (Fig. 3). The IGF I and IGF II protein tertiary structure have been predicted via Chimera (Fig. 4).

**Figure 5:**
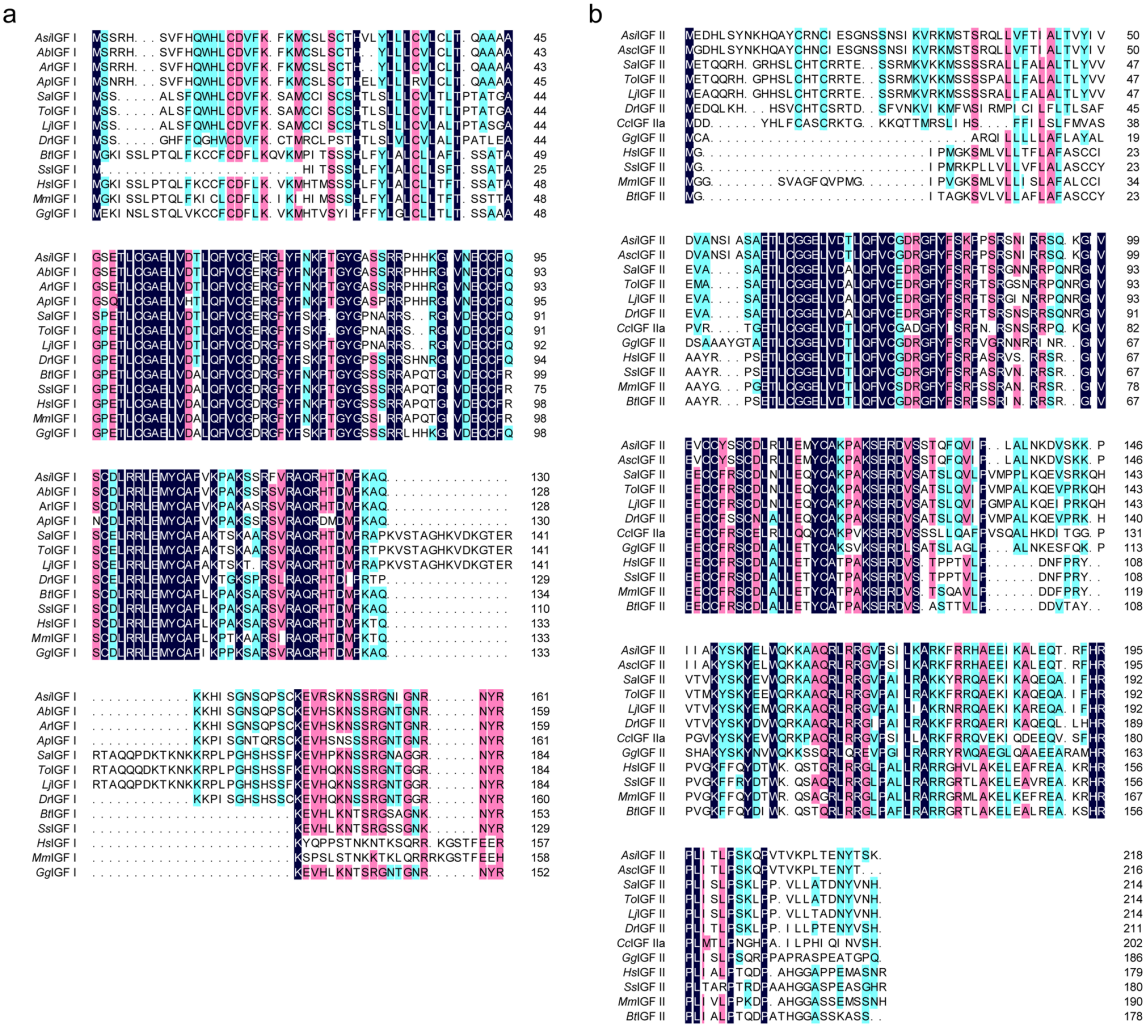
Multiple alignment of IGFs proteins in different species. The IGFs sequences from other species were downloaded from GenBank. The references for the GenBank Accession Numbers of IGFs amino acid sequences are as follows: *Acipenser sinensis* IGFs (*Asi*IGFs, MK028132 and MK028133); *Acipenser ruthenus* IGF I (*Ar*IGF I, DQ329352); *Acipenser baerii* IGF I (*Ab*IGF I, FJ428828); *Acipenser persicus* IGF I (*Ap*IGF I, GU325629); *Acipenser schrenckii* IGF II (*Asc*IGF II, KC484697); *Danio rerio* IGFs (*Dr*IGFs, NM_131825 and AY049027); *Lateolabrax japonicas* IGFs (*Lj*IGFs, JN596878 and JN596879); *Sparus aurata* IGFs (*Sa*IGFs, AY996779 and AY996778); *Trachinotus ovatus* IGFs (*To*IGFs, KT727922 and KT727923); *Gallus gallus* IGFs (*Gg*IGFs, NM_001004384 and NM_001030342); *Bos Taurus* IGFs (*Bt*IGFs, NM_001077828 and NM_174087); *Sus scrofa* IGFs (*Ss*IGFs, NM_214256 and XM_021080648); *Homo sapiens* IGFs (*Hs*IGFs, NM_001111283 and NP_001121070); *Mus musculus* IGFs (*Mm*IGFs, AY878193 and M14951.1) and *Cyprinus carpio* IGF II (*Cc*IGF II, HM641129). (**a**) Chinese sturgeon IGF I. (**b**) Chinese sturgeon IGF II.

To further understand the sequence similarities, the IGF I and IGF II of Chinese sturgeon were compared with those from other vertebrates using NCBI (https://blast.ncbi.nlm.nih.gov/Blast.cgi?PROGRAM=blastn&PAGE_TYPE=BlastSearch&LINK_LOC=blasthome). The sequence of IGF I exhibited higher similarity to fish species like *Huso huso* (97%), *Acipenser baerii* (96%), *Acipenser schrenckii* (98%), *Acipenser ruthenus* (96%), *Acipenser persicus* (95%). Similarly, IGF II sequence exhibited higher similarity with the fish species like *Acipenser schrenckii* (99%), *Scaphirhynchus platorynchus* (98%). Our multiple sequence alignments shows that Chinese sturgeon IGFs has high sequence identity to IGFs from other (Fig. 5).

**Figure 6:**
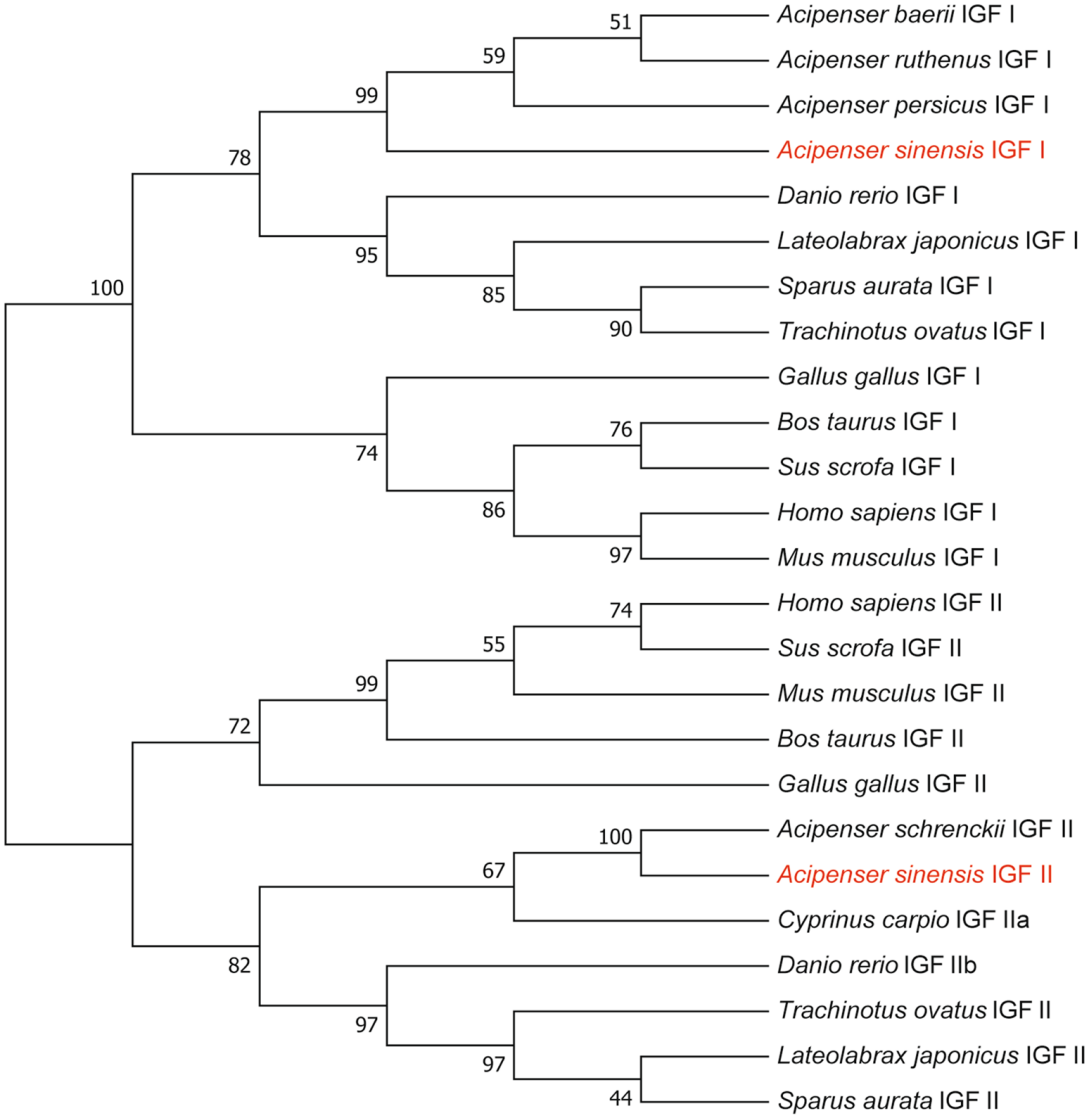
Phylogenetic tree of IGF I and IGF II constructed by the neighbor-joining method.

To evaluate the evolutionary relationships between the predicted Chinese sturgeon IGFs and other species IGFs, a phylogenetic tree was constructed (Fig. 6). There are two distinct lineages in this phylogenetic tree, one was composed of IGF I, while another was composed of IGF II. The phylogenetic tree showed that the Chinese sturgeon IGF I was closely related to that of *Acipenser ruthenus*, which was also belongs to the *Acipenseriformes* family. Similarly, the Chinese sturgeon IGF II was closely related to that of *Acipenser schrenckii* IGF II, which was belongs to the *Acipenseriformes* family, too. And sturgeon IGFs formed a clade with other fish subgroups, did not grouped into a clade with *Gallus gallus*, *Sus scrofa*, *Bos Taurus*, *Mus musculus* and *Homo sapiens*. The phylogenetic relationships is consistent with the classification and evolutionary status for these species.

### Expression patterns of GH, GHR, IGF I and IGF II in various tissues

**Figure 7:**
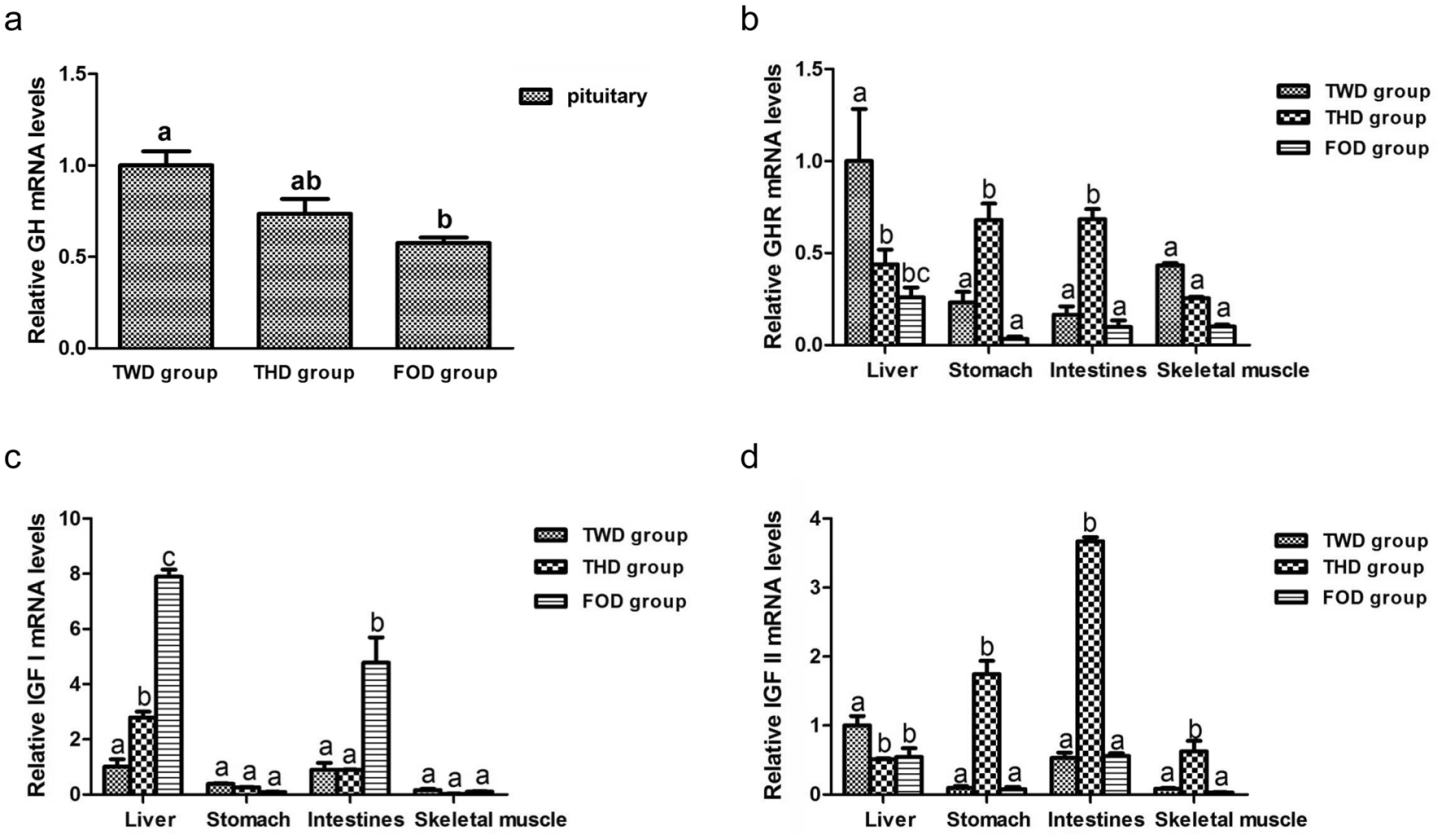
Response of GH (**a**), GHR (**b**), IGF I (**c**) and IGF II (**d**) to different feeding frequency in Chinese sturgeon. Data with different letters was significantly different (*p* < 0.05).

The relative expression of GH, GHR, IGF I and IGF II mRNA in various tissues of Chinese sturgeon were determined by quantitative real-time PCR. The relative expression of GH/IGF axis was significantly affected by feeding frequency (*p* < 0.05). The relative expression of GH in the pituitary decreased with increasing feeding frequency (Fig. 7). The relative expression of GHR in liver and skeletal muscle was deceased with increasing feeding frequency, while the relative expression of GHR in stomach and intestines at THD group was significantly higher than that of at TWD group and FOD group (*p* < 0.05) (Fig. 7). The relative expression of IGF I in liver increased significantly with increasing feeding frequency (*p* < 0.05) (Fig. 7). The relative expression of IGF I in stomach and skeletal muscle was similar at the three groups. The relative expression of IGF I in intestines was significantly higher at FOD group than at TWD group and THD group (*p* < 0.05). The relative expression of IGF II in liver at TWD group was significantly higher than that at THD group and FOD group (*p* < 0.05) (Fig. 7). However, the relative expression of IGF II in stomach, intestines and skeletal muscle at THD group was higher than that at TWD group and FOD group.

## Discussion

Growth is a major physiological process that is closely related in vertebrates. The GH/IGF axis is important in the neuroendocrine regulation of vertebrate growth^12,16^. The reasonable feeding frequency is an important factor for effective cost saving in intensive aquaculture. An appropriate feeding frequency can help the growth and metabolism of fish. Therefore, it is important to determine the optimum feeding frequency for the healthy and sustainable development of fish. In this study, the effects of different feeding frequencies on the growth and the expression of genes in the GH/IGF axis were assessed in juvenile Chinese sturgeon. The significant variation in the expression of genes related to the GH/IGF axis of juvenile Chinese sturgeon among the different feeding frequencies is interesting.

We first cloned and analyzed the IGF I and IGF II sequences from Chinese sturgeon. The complete coding region of IGF I from Chinese sturgeon encoded a putative protein composed of 162 amino acids with 45amino acid signal peptide. The result is similar to those of the Persian sturgeon^51^. The complete coding region of IGF II from Chinese sturgeon encoded a putative protein composed of 219 amino acids with no signal peptide. However, the IGF II from Amur sturgeon contained 47 amino acid signal peptide^52^. A multiple sequence alignment was constructed indicating that Chinese sturgeon IGFs display relatively high sequence identity comparing to those of other teleosts. The phylogenetic analysis of the full length amino acid sequences revealed that IGFs sequences from Chinese sturgeon have high similarity with IGFs from other species in the Acipenseriformes group. The result of phylogenetic analysis for the IGFs is consistent with the traditional taxonomy, and this result is consistent with previous study^53^. Those result suggested that the structure and function of IGFs of Chinese sturgeon share a higher degree of evolutionarily conserved in amino acid sequence.

The GH/IGF axis is influenced by environmental conditions and nutrition^50,54,55^. The GH was detected in the pituitary of Chinese sturgeon^56^. Interestingly, the expression of GH was showed lower levels at FOD group than that at TWD group and THD group at the end of the 38-day experiment in the present study. The GH is implicated in somatic growth and developmental processes. The higher expression of GH is in the fast-growing females *Cynoglossus semilaevis* and *Anguilla anguilla* than in males^57,58^. The expression levels of GH are decreased with fasting conditions in *Rhamdia quelen*^55^. The lower expression levels of GH may lead to the reduced growth of Chinese sturgeon at the higher feeding frequency group.

The GHR can mediate the biological actions of GH^17^. The binding of GH to GHR leads to the rapid activation of some signal transduction pathways that regulate cell growth. The expression levels of GHR can be affected by environmental factors in fish^59,60^. In this study, the expression levels of GHR were significantly affected by the feeding frequency. The expression levels of GHR decreased in the liver and skeletal muscle along with the increasing of feeding frequency. The muscle GHR of Atlantic salmon was elevated after fed high plant protein, while GH-IGF system had minor effects^61^. The GH had been reported to be participation in regulating GHR expression in fish^62,63^. In the liver and skeletal muscle, the expression levels of GHR are decreasing along with the increasing feeding frequency that is likely to be because of the decreasing of the expression levels of GH in the present study. And this is consistent with the theory that the expression level of GHR decreases as the expression level of GH decreases. However, the expression levels of GHR in the stomach and intestines are highest at THD group. This suggested it could up-regulated GHR expression in the stomach and intestines at the feeding frequency of three times daily.

In this study, the IGF I and IGF II were expressed differentially due to feeding frequency, indicating that the Chinese sturgeon IGF I and IGF II paly different roles in response to different feeding frequencies. The IGFs play an important role in somatic growth of vertebrates. In the present study, we observed that the expression levels of IGF I in the liver and intestines at FOD group was highest significantly among the three experimental groups. The IGF I increase in liver and intestines may support IGF I to be involved in the metabolism when fed four times daily compare to two and three times daily. There is no significantly difference to the expression levels of IGF I in the stomach and skeletal muscle among the three experiments. The expression levels of IGF II in the stomach, intestines and skeletal muscle at THD group were highest significantly among the three experimental groups. The expression levels of IGF II at TWD group were highest significantly in the liver.

Considering the relationship of GH, GHR and IGFs, the increasing expression levels of GHR can enhance GH sensitivity and increase the IGFs production. In opposite, the increasing expression levels of IGFs can reduce expression of GH production because of negative feedback. The increasing expression levels of IGF I in the liver and the decreasing expression levels of GH in this study support this hypothesis. Interestingly, no IGF I responses were detected in stomach and skeletal muscle at the different feeding frequency in this study. The current GHR response in the liver and skeletal muscle seems to be related to the decreasing of GH at three experiments. The decreasing GH may lead the low expression of GHR in the stomach and intestines at FOD group. Requeni et al.^64^ reported that the GH levels of rainbow trout were rose after feeding more plant protein, which maybe because of the decreasing of the IGF I. Confinement stress caused a significant decline in circulating IGF I after 3 h, and IGF II after 24 h in rainbow trout. However, confinement stress had little effect on the circulating GH in rainbow trout^48^. However, it cannot be adequately determined here whether the decline in GH is attributed solely to the increasing of IGF I in liver. More research is required to fully understand this mechanism.

The feeding frequency plays an important role in intensive aquaculture. An inappropriate feeding frequency can negatively affects the growth of fish. Higher feeding frequency can input costs and the too low feeding frequency can cause greater variety in fish size^10^. Therefore, it is important to confirm the optimum feeding frequency. Previous study about juvenile *Brazilian sardine*^65^ have suggested that feeding frequency had significant effects on biometric index of growth, such as the body weight gain, specific growth rate and feed efficiency rate. Increased feeding frequency has been reported to promote the growth of many fish species^66,67^. A lot of studies had been reported that the growth rate was increasing with feeding frequencies in many juvenile fish species^68,69^. These studies have suggested that feeding frequency had significant effects on weight gain and feeding behaviour of cultivated fish. Unfortunately, we could not compare the outcomes to other studies as no data on the expression levels of GH/GHR axis in fish under different feeding frequencies. As the frequency of feeding increases, the individual size difference decreases in rock bream^70^. Manecas Baloi et al.^65^ reported that the effective feeding frequency for good performance in farmed fish was at least twice daily. In contrast of fed only once daily, fish gained more weight under feeding more than once daily. Biswas et al.^71^ reported that feeding three times daily gained more weight and grew more efficiently for *Lates calcarifer* compared to one, two and four times daily. Atlantic spadefish grew more and more efficiently when feeding three times daily compared to once daily, regardless of feeding rate^72^. Mihelakakis et al.^73^ reported that juvenile common Pandora could obtain good growth and feed efficiency when were fed to satiation twice daily compared to one, three and four feeding daily. The optimal feed efficiency for growth performance of juvenile dark-banded Rockfish treated was 2 and 3 meals/d^74^. The optimal feeding frequency of captive head-started green turtles was feeding twice daily^75^. However, the feeding frequency had little effect on the growth of the tilapia^76^. Although the optimal feeding frequency differs according to the size and species of fish, increased feeding frequency may help increase the opportunity to access to feed and reduce the dominance in culture tank in fish. That might be because juvenile fish need to distribute more energy for competing tours to flock at the lower feeding frequency, which could accelerate the metabolism of lipid and protein. After 38 days of growth, the three different feeding frequencies groups of Chinese sturgeon had no significant difference of body length, body weight, specific growth rate, the survival rate, the rate of weight gain and the condition factor in this study. These changes do not appear to be consistent with the expression of GH/IGF axis. These is no significant difference of special growth rate with the increasing of feeding frequency in *A. schrenckii* Brandt♀ × *A. baeri* Brandt♂hybrid sturgeon for feeding 12 weeks, though special growth rate grows continuously with the increasing of feeding frequency^77^. That result is similar to our study. We suppose the experiment period was not long enough. The biometric index should be consistent with the expression of GH/IGF axis. This hypothesis need further studies to be verified. In our study, stocking density was not a limiting factor during the experiment, because the tank is large enough to meet the experiment requirements. No fish died in the three different feeding frequencies group during the 38 days of experiment. This result is similar to the previous studies^77^. 400 juvenile individuals of *A. schrenckii* Brandt♀ × *A. baeri* Brandt♂hybrid sturgeon (initial body weight 7.79 ± 0.37 g) were fed at three feeding frequencies for 12 weeks, and all fish survived throughout the trial^77^.

The significant difference in the expression of GH/IGF axis was found in different feeding frequencies in the present study. The process of growth is primarily governed by the GH/IGF axis. The IGFs are the primary mediators of the growth-promoting effects of GH. In the present study, we observed that the expression levels of IGF I in the liver at FOD group was highest significantly among the three experimental groups. Stress caused by a high feeding frequency may enhance the reallocation of metabolic energy. However, there is no significant difference of body length, body weight, specific growth rate, the survival rate, the rate of weight gain and the condition factor in the three different feeding frequencies groups. Further studies are necessary to investigate the specific mechanism by which GH/IGF axis transcription alters growth performance in Chinese sturgeon. In addition, considering the relationship among IGFs, GH, and GHR, the increasing expression levels of IGFs can reduce expression of GH production because of negative feedback. Many studies have proved that most transcripts are produced in the liver under the stimulation of GH^78,79^. The increasing expression levels of IGF I in the liver and the decreasing expression levels of GH in this study are consistent with this hypothesis. The growth-promoting effects of GH was mediated by IGFs. Systemic IGF I can act on target tissues to promote cell differentiation, proliferation and ultimately body growth^80^. IGF I has been extensively researched as a candidate gene for growth improvement in many species. The IGF I gene have been proved to have a direct role in the determination of growth rate in cattle breed^81,82^. Polymorphisms at the IGF I gene are considered to be a possible QTL marker for birth weight in pig^83^. Interestingly, an increasing of IGF I in liver was observed with the increasing of feeding frequency in this study, a result which may have potentially been caused by increasing of blood supply to the liver. Plasma IGF I and liver IGF I mRNA levels have been reported to have positive and significant correlations in juvenile rainbow trout (*Oncorhynchus mykiss*)^84^, juvenile southern flounder (*Paralichthys lethostigma*)^85^, and in juvenile hybrid striped bass (*Morone chrysops* × *Morone saxatalis*)^86^. The increasing of blood supply to the liver had previously been identified as the major source of circulating IGF I^45^. The somatic growth and plasma IGF I in vertebrates had been reported to have a positive correlation^60^. Furthermore, the previous study have reported that liver IGF I mRNA levels were correlated to growth rate in juvenile tilapia^87,88^. Although this needs to be proven in all species and there is still issues to be note that the appropriate normalization of liver IGF I mRNA levels were essential to accurately reflect biological significance^89^. And there is sufficient data extant to suggest that measures of IGF I mRNA levels can be a helpful method for growth of fish over precisely defined and physiologically structured groups in both cultured and natural conditions^16^. Based on the case of IGF I have the direct contributions to regulate somatic growth, along with the correlations with growth rate in fish, IGF I may be a candidate gene to measure instantaneous growth, although the biomarkers for growth can be both situation specific and species^89^. As feeding frequency is relation with cost involved, optimal feeding frequency should be a cost-effective management approach during fish growth. The expression level of IGF I is regarded as a biomarker of growth performance in fish^90^. The increasing expression levels of IGF I in liver at increased feeding frequencies obtained in our study agree with the results reported by the researchers cited above. Therefore, based on these previous studies that liver IGF I is regarded as a biomarker of growth performance, it is better for growth when feeding four times daily compared to twice and thrice daily.

Overall, in the present study we have observed clear effects of feeding frequency on the expression of GH, GHR, IGF I and IGF II. The significant difference in the expression of GH/IGF axis was found in different feeding frequencies in the present study. Further studies investigating the characterized relationship between GH and IGFs, their respective receptor expression levels and circulating concentrations, are required to fully understand. The three different feeding frequencies groups had no significant difference of body length, body weight, specific growth rate, the survival rate, the rate of weight gain and the condition factor in only 38 days of feeding for juvenile Chinese sturgeon. An increasing of IGF I in liver was observed with the increasing of feeding frequency in this study. According to the previous study, the expression level of IGF I is regarded as a biomarker of growth performance in fish^90^. Therefore this result suggested that the juvenile Chinese sturgeon is better for growth when feeding four times daily compared to twice and thrice daily.

## Conclusion

In conclusion, we have confirmed that there were significant correlations between feeding frequency and expression of GH/IGF axis in this experiment. However, the preliminary nature of this work must be acknowledged.

The full-length of the Coding Sequences of IGF I and IGF II genes (489-bp and 660-bp, respectively), were cloned and analyzed in Chinese sturgeon the first time. Multiple sequence alignments of IGFs revealed that Chinese sturgeon are high sequence identity to IGFs from other species. IGFs were detected in the four tissues (liver, stomach, intestines and skeletal muscle) of Chinese sturgeon. In this study, the expression of GH/IGF axis was significantly affected by feeding frequency. The relative expression of Chinese sturgeon IGF I in liver increased significantly with increasing feeding frequency (*p* < 0.05) in this study. Based on these previous studies that liver IGF I is regarded as a biomarker of growth performance, this result suggested that the juvenile Chinese sturgeon is better for growth when feeding four times daily compared to twice and thrice daily.

## Data Availability

Data are available from the corresponding author upon reasonable request.

## References

[1] Peng Z (2007). Age and biogeography of major clades in sturgeons and paddlefishes (Pisces: Acipenseriformes). Mol. Phylogenet. Evol..

[2] Zhuang P (2002). Ontogenetic behavior and migration of Chinese sturgeon, *Acipenser sinensis*. Environ. Biol. Fishes.

[3] Wang H (2014). Light intensity preferences of 5-month and 7-month F_2 Chinese sturgeon (*Acipenser sinensis*). J. Fish. China.

[4] Ping Z (2016). New evidence may support the persistence and adaptability of the near-extinct Chinese sturgeon. Biol. Conserv..

[5] Cao H (2009). Molecular characterization of Chinese sturgeon gonadotropins and cellular distribution in pituitaries of mature and immature individuals. Mol. Cell. Endocrinol..

[6] Garcia JA, Villarroel M (2009). Effect of feed type and feeding frequency on macrophage functions in tilapia (*Oreochromis niloticus* L.). Fish Shellfish Immunol..

[7] Tucker BJ (2006). Effects of photoperiod and feeding frequency on performance of newly weaned Australian snapper *Pagrus auratus*. Aquaculture.

[8] Folkvord A, Otterå H (1993). Effects of initial size distribution, day length, and feeding frequency growth, survival, and cannibalism in juvenile Atlantic cod *Gadus morhua* L. Aquaculture.

[9] Biswas G, Jena JK, Singh SK, Patmajhi P, Muduli HK (2006). Effect of feeding frequency on growth, survival and feed utilization in mrigal, *Cirrhinus mrigala*, and rohu, *Labeo rohita*, during nursery rearing. Aquaculture.

[10] Ahmed I (2007). Effect of ration size on growth, body composition, and energy and protein maintenance requirement of fingerling Indian major carp, *Labeo rohita* (Hamilton). Fish Physiol. Biochem..

[11] Schnaittacher G, King W, Berlinsky DL (2015). The effects of feeding frequency on growth of juvenile Atlantic halibut, *Hippoglossus hippoglossus* L. Aquac. Res..

[12] Heras VDL, Martos-Sitcha JA, Yúfera M, Mancera JM, Martínez-Rodríguez G (2015). Influence of stocking density on growth, metabolism and stress of thick-lipped grey mullet (*Chelon labrosus*) juveniles. Aquaculture.

[13] Duan C (1997). The insulin-like growth factor system and its biological actions in fish. Integr. Comp. Biol..

[14] Fuentes EN, Valdés JA, Molina A, Björnsson BT (2013). Regulation of skeletal muscle growth in fish by the growth hormone—insulin-like growth factor system. Gen. Comp. Endocrinol..

[15] Salmerón C, Acerete L, Gutiérrez J, Navarro I, Capilla E (2013). Characterization and endocrine regulation of proliferation and differentiation of primary cultured preadipocytes from gilthead sea bream (*Sparus aurata*). Domest. Anim. Endocrinol..

[16] Beckman BR (2011). Perspectives on concordant and discordant relations between insulin-like growth factor 1 (IGF1) and growth in fishes. Gen. Comp. Endocrinol..

[17] Reindl KM, Sheridan MA (2012). Peripheral regulation of the growth hormone-insulin-like growth factor system in fish and other vertebrates. Comp. Biochem. Physiol. Part A.

[18] Tanamati F, Schuroff GP, Nascimento CSD, Vesco APD, Gasparino E (2015). GHR and IGF-I gene expression and production characteristics associated with GH gene polymorphism in Nile tilapia. Aquaculture.

[19] Peter RE, Marchant TA (1995). The endocrinology of growth in carp and related species. Aquaculture.

[20] Saltiel AR, Kahn CR (2001). Insulin signalling and the regulation of glucose and lipid metabolism. Nature.

[21] Taniguchi CM, Brice E, Kahn CR (2006). Critical nodes in signalling pathways: insights into insulin action. Nat. Rev. Mol. Cell Biol..

[22] Ayson FG (2002). Differential expression of insulin-like growth factor I and II mRNAs during embryogenesis and early larval development in rabbitfish *Siganus guttatus*. Gen. Comp. Endocrinol..

[23] Vong QP, Chan KM, Cheng CHK (2003). Quantification of common carp (*Cyprinus carpio*) IGF-I and IGF-II mRNA by real-time PCR: differential regulation of expression by GH. J. Endocrinol..

[24] Luckenbach JA, Murashige R (2007). Temperature affects insulin-like growth factor I and growth of juvenile southern flounder, *Paralichthys lethostigma*. Comp. Biochem. Physiol. A Mol. Integr. Physiol..

[25] Escobar S (2011). Molecular cloning of IGF-1 and IGF-1 receptor and their expression pattern in the Chilean flounder (*Paralichthys adspersus*). Comp. Biochem. Physiol. B: Biochem. Mol. Biol..

[26] Wood AW, Duan CH (2005). Insulin-like growth factor signaling in fish. Int. Rev. Cytol..

[27] Patiño R, Kagawa H (1999). Regulation of gap junctions and oocyte maturational competence by gonadotropin and insulin-like growth factor-I in ovarian follicles of red seabream. Gen. Comp. Endocrinol..

[28] Weber GM, Sullivan CV (2000). Effects of insulin-like growth factor-I on in vitro final oocyte maturation and ovarian steroidogenesis in striped bass, *Morone saxatilis*. Biol. Reprod..

[29] Loir M (2015). Spermatogonia of rainbow trout: II. In vitro study of the influence of pituitary hormones, growth factors and steroids on mitotic activity. Mol. Reprod. Dev..

[30] Pozios KC, Ding J, Degger B, Upton Z, Duan C (2001). IGFs stimulate zebrafish cell proliferation by activating MAP kinase and PI3-kinase-signaling pathways. Am. J. Physiol. Regul. Integr. Comp. Physiol..

[31] Scanes CG, Dunnington EA, Buonomo FC, Donoghue DJ, Siegel PB (1989). Plasma concentrations of insulin like growth factors (IGF-)I and IGF-II in dwarf and normal chickens of high and low weight selected lines. Growth Dev. Aging Gda.

[32] Beccavin C, Chevalier B, Cogburn LA, Simon J, Duclos MJ (2001). Insulin-like growth factors and body growth in chickens divergently selected for high or low growth rate. J. Endocrinol..

[33] Duan C, Plisetskaya EM (1993). Nutritional regulation of insulin-like growth factor-I mRNA expression in salmon tissues. J. Endocrinol..

[34] Jean-Charles G (2003). Effects of environmental temperature on IGF1, IGF2, and IGF type I receptor expression in rainbow trout (*Oncorhynchus mykiss*). Gen. Comp. Endocrinol..

[35] Davis ME, Simmen RCM (2006). Genetic parameter estimates for serum insulin-like growth factor I concentrations, and body weight and weight gains in Angus beef cattle divergently selected for serum insulin-like growth factor I concentration. J. Anim. Sci..

[36] Giorgi B, Jean-FranOis B, Elisabeth E, Manfred R (2010). Insulin-like growth factor-3 (IGF-3) in male and female gonads of the tilapia: development and regulation of gene expression by growth hormone (GH) and 17alpha-ethinylestradiol (EE2). Gen. Comp. Endocrinol..

[37] Carnevali O (2005). Hormonal regulation of liver IGF-I and IGF-II gene expression in the marine teleost *Sparus aurata*. Mol. Reprod. Dev..

[38] Pierce AL, Breves JP, Moriyama S, Hirano T, Grau EG (2011). Differential regulation of Igf1 and Igf2 mRNA levels in tilapia hepatocytes: effects of insulin and cortisol on GH sensitivity. J. Endocrinol..

[39] Shamblott MJ, Cheng CM, Bolt D, Chen TT (1995). Appearance of insulin-like growth factor mRNA in the liver and pyloric ceca of a teleost in response to exogenous growth hormone. Proc. Natl. Acad. Sci. U.S.A..

[40] Greene MW, Shamblott MJ, Chen TT (1999). Presence of GH-dependent IGF-II mRNA in the diffuse pancreatic tissue of a teleost. Comp. Biochem. Physiol. B: Biochem. Mol. Biol..

[41] Dechiara TM, Robertson EJ, Efstratiadis A (1991). Parental imprinting of the mouse insulin-like growth factor II gene. Cell.

[42] Kajimura S (2002). Effects of insulin-like growth factors (IGF-I and-II) on growth hormone and prolactin release and gene expression in euryhaline tilapia, *Oreochromis mossambicus*. Gen. Comp. Endocrinol..

[43] Moriyama S, Yamaguchi K, Takasawa T, Chiba H, Kawauchi H (2006). Insulin-like growth factor I of Japanese eel, *Anguilla japonica*: cDNA cloning, tissue distribution, and expression after treatment with growth hormone and seawater acclimation. Fish Physiol. Biochem..

[44] Reinecke M (2005). Growth hormone and insulin-like growth factors in fish: where we are and where to go. Gen. Comp. Endocrinol..

[45] Duan C (1998). Nutritional and developmental regulation of insulin-like growth factors in fish. J. Nutr..

[46] Moriyama S, Ayson FG, Kawauchi H (2000). Growth regulation by insulin-like growth factor-I in fish. J. Agric. Chem. Soc. Japan.

[47] Perrot V, Funkenstein B (1999). Cellular distribution of insulin-like growth factor II (IGF-II) mRNA and hormonal regulation of IGF-I and IGF-II mRNA expression in rainbow trout testis (*Oncorhynchus mykiss*). Fish Physiol. Biochem..

[48] Wilkinson RJ, Porter M, Woolcott H, Longland R, Carragher JF (2006). Effects of aquaculture related stressors and nutritional restriction on circulating growth factors (GH, IGF-I and IGF-II) in Atlantic salmon and rainbow trout. Comp. Biochem. Physiol. A Mol. Integr. Physiol..

[49] Small BC, Peterson BC (2005). Establishment of a time-resolved fluoroimmunoassay for measuring plasma insulin-like growth factor I (IGF-I) in fish: effect of fasting on plasma concentrations and tissue mRNA expression of IGF-I and growth hormone (GH) in channel catfish (*Ictalurus punctatus*). Domest. Anim. Endocrinol..

[50] Tu Y (2015). Growth performance, digestive enzyme, transaminase and GH-IGF-I axis gene responsiveness to different dietary protein levels in broodstock allogenogynetic gibel carp (*Carassius auratus gibelio*) CAS III. Aquaculture.

[51] Yarmohammadi M (2014). Persian sturgeon insulin-like growth factor I: molecular cloning and expression during various nutritional conditions. J. Appl. Genet..

[52] Ren Y, Wen H, Yun L, Li J (2017). Stocking density affects the growth performance and metabolism of Amur sturgeon by regulating expression of genes in the GH/IGF axis. Chin. J. Oceanol. Limnol..

[53] Ma Z (2016). Insulin-like growth factor (IGF) genes in golden pompano *Trachinotus ovatus* (Linnaeus 1758) larvae: molecular cloning and response to water temperature and nutrient manipulation. Israeli J. Aquacult. Bamidgeh.

[54] Hanson AM, Kittilson JD, Martin LE, Sheridan MA (2014). Environmental estrogens inhibit growth of rainbow trout (*Oncorhynchus mykiss*) by modulating the growth hormone-insulin-like growth factor system. Gen. Comp. Endocrinol..

[55] Menezes C (2015). The influence of stocking density and food deprivation in silver catfish (*Rhamdia quelen*): a metabolic and endocrine approach. Aquaculture.

[56] Cao H, Zhou RJ, Wei QW, Li CJ, Gui JF (2011). Molecular characterization of the growth hormone in Chinese sturgeon and its expression during embryogenesis and early larval stages. J. Appl. Ichthyol..

[57] Qian M (2012). Genomic structure, polymorphism and expression analysis of the growth hormone (GH) gene in female and male Half-smooth tongue sole (*Cynoglossus semilaevis*). Gene.

[58] Degani G, Tzchori I, Yom-Din S, Goldberg D, Jackson K (2003). Growth differences and growth hormone expression in male and female European eels [*Anguilla anguilla* (L.)]. Gen. Comp. Endocrinol..

[59] Poppinga J, Kittilson J, Mccormick SD, Sheridan MA (2007). Effects of somatostatin on the growth hormone-insulin-like growth factor axis and seawater adaptation of rainbow trout (*Oncorhynchus mykiss*). Aquaculture.

[60] Peterson BC, Small BC (2005). Effects of exogenous cortisol on the GH/IGF-I/IGFBP network in channel catfish. Domest. Anim. Endocrinol..

[61] Sissener NH (2013). Effects of plant-based diets on glucose and amino acid metabolism, leptin, ghrelin and GH-IGF system regulation in Atlantic salmon (*Salmo salar* L). Aquac. Nutr..

[62] Baowei J (2006). The co-existence of two growth hormone receptors in teleost fish and their differential signal transduction, tissue distribution and hormonal regulation of expression in seabream. J. Mol. Endocrinol..

[63] Very NM, Sheridan MA (2007). Somatostatin regulates liver growth hormone sensitivity by internalizing growth hormone receptors and by decreasing transcription of growth hormone receptor mRNAs. Am. J. Physiol. Regul. Integr. Comp. Physiol..

[64] Pedro GR (2005). Regulation of the somatotropic axis by dietary factors in rainbow trout (*Oncorhynchus mykiss*). Br. J. Nutr..

[65] Baloi M, Carvalho CVAD, Sterzelecki FC, Passini G, Cerqueira VR (2016). Effects of feeding frequency on growth, feed efficiency and body composition of juveniles Brazilian sardine, *Sardinella brasiliensis* (Steindacher 1879). Aquac. Res..

[66] Wang N, Hayward RS, Noltie DB (1998). Effect of feeding frequency on food consumption, growth, size variation, and feeding pattern of age-0 hybrid sunfish. Aquaculture.

[67] Lee SM, Cho SH, Kim DJ (2015). Effects of feeding frequency and dietary energy level on growth and body composition of juvenile flounder, *Paralichthys olivaceus* (Temminck & Schlegel). Aquac. Res..

[68] Dong GF (2015). Individual variations and interrelationships in feeding rate, growth rate, and spontaneous activity in hybrid tilapia (*Oreochromis niloticus* × *O. aureus*) at different feeding frequencies. J. Appl. Ichthyol..

[69] Zhou Z (2010). Effect of feeding frequency on growth, feed utilization, and size variation of juvenile gibel carp (*Carassius auratus gibelio*). J. Appl. Ichthyol..

[70] Oh SY, Maran BAV (2015). Feeding frequency influences growth, feed consumption and body composition of juvenile rock bream (*Oplegnathus fasciatus*). Aquacult. Int..

[71] Biswas G, Thirunavukkarasu AR, Sundaray JK, Kailasam M (2010). Optimization of feeding frequency of Asian seabass (*Lates calcarifer*) fry reared in net cages under brackishwater environment. Aquaculture.

[72] Trushenski J (2012). Feeding rate and frequency affect growth of juvenile Atlantic spadefish. N. Am. J. Aquac..

[73] Mihelakakis A (2001). Effect of feeding frequency on growth, feed efficiency, and body composition in young common pandora. Aquac. Int..

[74] Oh SY, Maran BAV, Jin WP (2018). Effect of feeding frequency on growth, food consumption, proximate composition, and blood chemistry of juvenile dark-banded rockfish, *Sebastes inermis*. J. World Aquac. Soc..

[75] Kanghae H (2016). Optimal feeding frequency of captive head-started green turtles (*Chelonia mydas*). J. Anim. Physiol. Anim. Nutr..

[76] Huang Q (2015). Feeding frequency and rate effects on growth and physiology of juvenile genetically improved farmed Nile Tilapia. N. Am. J. Aquac..

[77] Luo L (2015). Effects of feeding rates and feeding frequency on the growth performances of juvenile hybrid sturgeon, *Acipenser schrenckii* Brandt♀ × *A. baeri* Brandt. Aquaculture.

[78] Carnevali O (2005). Hormonal regulation of hepatic IGF-I and IGF-II gene expression in the marine teleost *Sparus aurata*. Mol. Reprod. Dev..

[79] Pell JM, Saunders JC, Gilmour RS (1993). Differential regulation of transcription initiation from insulin-like growth factor-I (IGF-I) leader exons and of tissue IGF-I expression in response to changed growth hormone and nutritional status in sheep. Endocrinology.

[80] Wood AW (2005). Insulin-like growth factor signaling in fish. Int. Rev. Cytol..

[81] Curi RA (2005). Association between IGF-I, IGF-IR and GHRH gene polymorphisms and growth and carcass traits in beef cattle. Livest. Prod. Sci..

[82] Pereira A (2005). Association of GH and IGF-1 polymorphisms with growth traits in a synthetic beef cattle breed. Genet. Mol. Biol..

[83] Casas-Carrillo EP (2015). Relationship of growth hormone and insulin-like growth factor-1 genotypes with growth and carcass traits in swine. Anim. Genet..

[84] Gabillard JC (2003). Effects of environmental temperature on IGF1, IGF2, and IGF type I receptor expression in rainbow trout (*Oncorhynchus mykiss*). Gen. Comp. Endocrinol..

[85] Luckenbach JA (2007). Temperature affects insulin-like growth factor I and growth of juvenile southern flounder, Paralichthys lethostigma. Comp. Biochem. Physiol. Part A Mol. Integr. Physiol..

[86] Picha ME (2008). Regulation of endocrine and paracrine sources of Igfs and Gh receptor during compensatory growth in hybrid striped bass (*Morone chrysops* × *Morone saxatilis*). J. Endocrinol..

[87] Kajimura S (2001). Stimulation of insulin-like growth factor-I production by recombinant bovine growth hormone in Mozambique tilapia, *Oreochromis mossambicus*. Fish Physiol. Biochem..

[88] Cruz EMV (2006). Insulin-like growth factor-I cDNA cloning, gene expression and potential use as a growth rate indicator in Nile tilapia, *Oreochromis niloticus*. Aquaculture.

[89] Picha ME (2008). Endocrine biomarkers of growth and applications to aquaculture: a minireview of growth hormone, insulin-like growth factor (IGF)-I, and IGF-binding proteins as potential growth indicators in fish. N. Am. J. Aquac..

[90] Picha ME (2014). Overcompensation of circulating and local insulin-like growth factor-1 during catch-up growth in hybrid striped bass (*Morone chrysops* × *Morone saxatilis*) following temperature and feeding manipulations. Aquaculture.

